# Alerting or Somnogenic Light: Pick Your Color

**DOI:** 10.1371/journal.pbio.2000111

**Published:** 2016-08-15

**Authors:** Patrice Bourgin, Jeffrey Hubbard

**Affiliations:** 1 CNRS-UPR 3212, Institute of Cellular and Integrative Neurosciences, Sleep Disorders Center—CIRCSom, CHU and FMTS, University of Strasbourg, Strasbourg, France; 2 Center for Integrative Genomics, University of Lausanne, Lausanne, Switzerland

## Abstract

In mammals, light exerts pervasive effects on physiology and behavior in two ways: indirectly through clock synchronization and the phase adjustment of circadian rhythms, and directly through the promotion of alertness and sleep, respectively, in diurnal and nocturnal species. A recent report by Pilorz and colleagues describes an even more complex role for the acute effects of light. In mice, blue light acutely causes behavioral arousal, whereas green wavelengths promote sleep. These opposing effects are mediated by melanopsin-based phototransduction through different neural pathways. These findings reconcile nocturnal and diurnal species through a common alerting response to blue light. One can hypothesize that the opposite responses to natural polychromatic light in night- or day-active animals may reflect higher sensitivity of nocturnal species to green, and diurnals to blue wavelengths, resulting in hypnogenic and alerting effects, respectively. Additional questions remain to be clarified. How do different light wavelengths affect other behaviors such as mood and cognition? How do those results apply to humans? How does light pose either a risk or benefit, depending on whether one needs to be asleep or alert? Indeed, in addition to timing, luminance levels, and light exposure duration, these findings stress the need to understand how best to adapt the color spectrum of light to our needs and to take this into account for the design of daily lighting concepts—a key challenge for today’s society, especially with the emergence of LED light technology.

## Introduction

Human beings, along with nearly all other mammals on Earth, are programmed to live under a daily light–dark cycle generated by our planet’s rotation on its axis relative to the sun. During the course of one 24-hour day, we are exposed to different colors of light, which is a function of wavelengths on the visible spectrum. Indeed, we commonly think of light as a primarily visual medium, our way for perceiving the world around us. However, light exposure serves another function, affecting a large range of physiological and behavioral parameters, including sleep and alertness. With the introduction and development of artificial light, we have progressively changed our lifestyle, extending our daylight hours beyond nature, to provide an environment conducive to self-fulfillment, and subconsciously exposing ourselves repeatedly to luminance pollution. This phenomenon has been exacerbated with the boom in new light-emitting technologies such as smartphones and tablets. Unfortunately, there are detrimental costs, resulting in the impairment of the sleep–wake architecture, which leads to an increased incidence of circadian disorders, insomnia, daytime sleepiness, mood alteration, and poorer cognitive performance [1,2]. Little attention has been paid to the non-visual influence of light until the more recent discovery and emerging knowledge about melanopsin. This photopigment, which is maximally sensitive to blue light, has turned out to be a key player involved in the non-image forming effects of light [3–5]. As a consequence of this growing research interest, there is now a large body of evidence that reveals a deeper and more complex role of light on brain activity and behavior than previously thought. However, critical questions remain to be clarified, such as how the photic regulation of sleep and waking depend on the time of day, the duration and intensity of light exposure, and perhaps most interestingly, the spectral composition of light, an aspect specifically addressed by Pilorz et al. [6] in the present issue of *PLOS Biology*.

## Light and Melanopsin: Role in Regulation of Sleep and Alertness

Photoreception in the mammalian retina is not restricted to rods and cones, the “classical” photoreceptors that generate an image of the world, but extends to melanopsin (OPN4), a third photoreceptor which also provides a measurement of environmental brightness (irradiance). Melanopsin is expressed in a subset of retinal ganglion cells (ipRGCs) that convey all non-visual light information, from melanopsin and rod/cone-based photodetection, to the brain [7]. More specifically, the ipRGCs consist of different subpopulations that have distinct brain targets, schematically classified as M1, innervating the suprachiasmatic nucleus where the primary clock is located, and non-M1, with projections to several brain areas outside the suprachiasmatic nuclei (SCN) [8,9]. Consecutive to this anatomical distribution, the photic signals modulate sleep and waking in two different ways: either indirectly, through a well-characterized circadian pathway, or directly, through less well-understood mechanisms [10]. Light entrains the clock to adjust the phase of circadian rhythms and synchronize the sleep–wake pattern to the time of day. Independently of the circadian system, light also exerts a direct influence, acutely promoting sleep in nocturnal animals and increasing alertness in diurnal species [10,11]. Light also influences the response to sleep-loss [5].

In recent years, the development of transgenic mouse models targeting different components of the phototransduction pathways has provided important insights into the mechanisms underlying the photic regulation of sleep (for review, see [10]). In mice, light readily induces sleep, whereas dark pulses promote wakefulness. We, and others, have demonstrated to what extent the alteration of light input to the brain by genetic ablation or inhibition of phototransduction components (rods and cones, *Opn4* or ipRGCs), affects vigilance states [3–5]. Collectively, these studies suggest that the effects of light and darkness, respectively promoting sleep and waking, are primarily mediated by melanopsin-based phototransduction, yet rods and cones also play a role. Furthermore, the OPN4-dependent effect of light was correlated with an activation of galanin-containing “sleep-promoting” neurons in the ventrolateral preoptic area (VLPO) at the hypothalamic level, therefore identifying a pathway, independent from the SCN, by which light regulates sleep [4,5,10]. Moreover, these studies also defined how different parameters such as intensity, duration, and time of day of light exposure can affect this response. Thus, we previously showed, in mice, that the photic regulation of sleep and waking is influenced by the time of day, with light and dark effects primarily mediated by melanopsin-based phototransduction during the dark phase and rods/cones during the light phase, suggesting that both phototransduction systems compensate for each other depending on circadian phase [5]. Moreover, the degree of sleep response is dependent upon the intensity [4,12] and duration of the stimulus, indicating a role for melanopsin in sleep maintenance [13]. Furthermore, we previously observed that mice lacking melanopsin sleep 1 hour less during the light phase of a 24-hour cycle, suggesting that light additionally exerts sustained effects over a longer period of time [5]. However, little attention has been paid to the influence of the color spectrum of light on sleep and waking.

In humans there is now a large body of evidence that exposure to light directly enhances alertness and performance, in essence regulating wakefulness and cognition [11,14]. More specifically, this is supported by different measures of alertness correlates such as self-rated scales, psychomotor vigilance tasks or higher cognitive tasks, and EEG or functional brain imagery (PET or fMRI recordings), during which the subjects perform non-visual cognitive tasks. A recent series of neuroimaging studies aimed to identify how brain activity related to ongoing auditory tasks is modulated by light exposure [14]. All studies converge to show that blue-enriched light is more efficient in increasing performance and decreasing sleepiness, suggesting a primary mediation through melanopsin-based phototransduction. This is consistent with findings that light stimulates cognitive brain activity in visually blind subjects who retain non-visual photoreception, but no functional rod/cone-based photoreception [15]. Moreover, this conclusion is corroborated by the observation that the direct influence of light on alertness, vigilance, the waking EEG, and executive brain responses depends on prior light history [16,17]. This latter observation is coherent with recent fundamental work suggesting that bistability properties of the protein with two different states may confer a “photic memory” responsible for slightly different responses to light dependent on previous light exposure [18]. The alerting effect of light in humans is also dependent on the luminance level [19] and duration [11], with fMRI studies reporting that increased exposure time and/or higher intensities trigger larger and longer lasting modulations of task-related responses [14]. The time of day also influences these alerting properties of light, though more studies were conducted in the evening or at night than in the morning or at midday. Interestingly, many investigations have also occurred under daily living conditions. For example, enrichment in blue light in an office environment facilitates alertness, higher performance, and higher sleep quality during the following night [20]. In the hours prior to bedtime, reading a book on a light-emitting device (LE-eBook), compared to reading print, increases evening alertness, thus reducing sleep latency [21], an effect which is attenuated when using blue blocker glasses [22]. This latter work underlines the potential benefit of investigating the influence of light wavelength on the alerting response. Overall, the effects are stronger with blue (460–480 nm) as opposed to green (555 nm) monochromatic light, in promoting alertness during both day and night [23], waking EEG [23,24], or task-related brain responses [14].

## Blue Versus Green: The Duality of Light’s Color Spectrum

**Fig 1:**
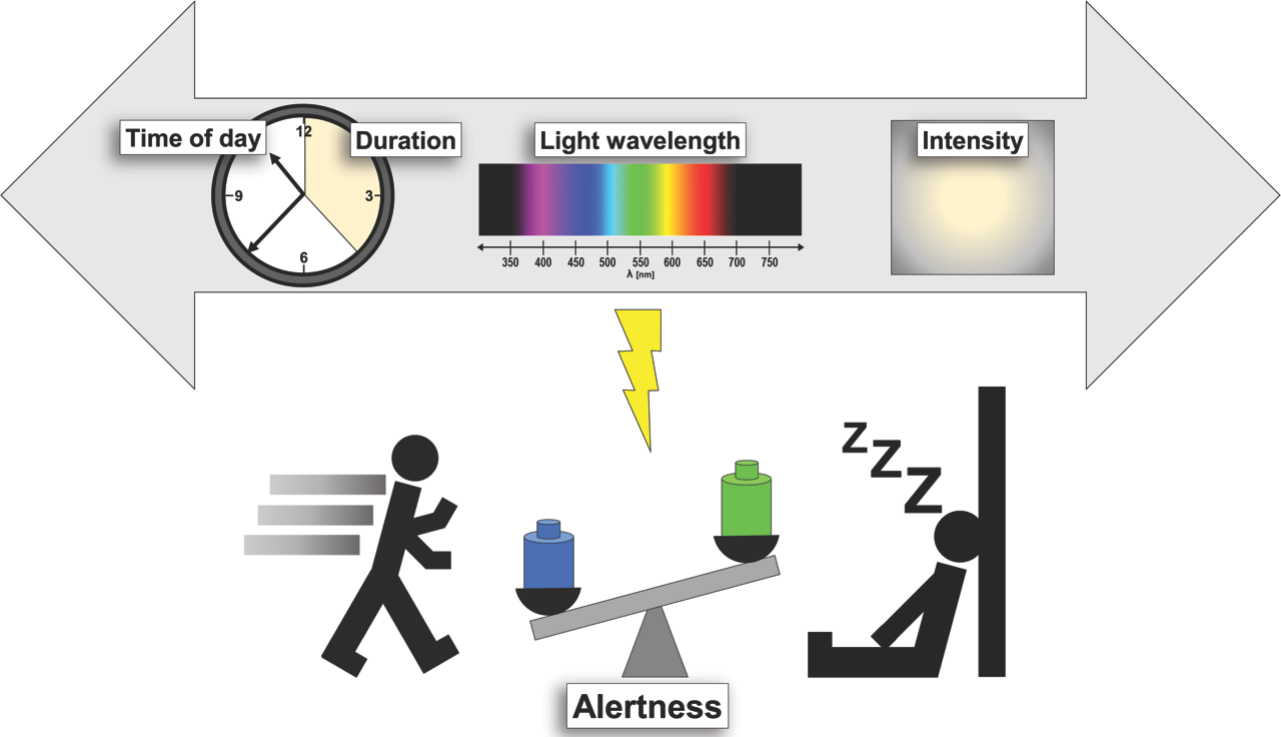
Summary of light parameters influencing sleep and alertness in humans. In humans, light exerts strong alerting effects that depend upon several parameters, such as dose (irradiance), duration, and the time of day of light exposure. This photic regulation of alertness also critically depends on the spectral composition of light, with blue light as a powerful alerting stimulus, contrary to green light, which might be less powerful to awaken a subject from sleep.

Although previous research has elucidated how timing, luminance levels, and exposure duration to light influence a somnogenic response in mice and an alerting one in humans, surprisingly few studies have focused on the role of narrow wavelength bands (colors) within the visible spectrum. In the current issue of *PLOS Biology*, Pilorz and colleagues investigated in wild-type and *Opn4*^*-/-*^ mice the effects of light at different wavelengths on sleep and waking, and corresponding changes in behavior as well as adrenal corticosterone release and molecular responses. To achieve this, they applied photic pulses of three different wavelengths, violet (405 nm), blue (470 nm), and green (530 nm), to distinguish differential activation of ultraviolet-sensitive (UVS) cones, melanopsin, and rods/midwave-sensitive (MWS) cones, respectively. One fascinating observation they report is that light can exert opposing effects on sleep and arousal that are dependent upon wavelength and are both melanopsin-mediated, but via different neural pathways. These data show that blue light promotes alertness, whereas green light induces sleep, as indexed by a video-tracking method previously validated for sleep studies. To further characterize their findings, it would be interesting to determine whether green light also improves sleep quality and continuity, and how it differentially modulates non-rapid eye movement (NREM) and rapid eye movement (REM) sleep, questions answerable with EEG recordings. Another immediate logical step is to determine how blue-related arousing effects correlate to cognitive performance, and whether different wavelengths impact other aspects such as depression-like behavior, as mood is known to be strongly influenced by light [2]. One more immediate challenge to investigate is how the effects of blue and green wavelengths interact with intensity, duration, and time of day of exposure (Fig 1).

Another innovative aspect of the study is the administration of the photic stimuli in *Opn4*^*-/-*^ mice, which shows that the effects of both blue and green wavelengths are reduced in the absence of melanopsin. This observation is counterintuitive and suggests a more complex role for melanopsin than originally thought. Indeed, the somnogenic influence of white light in mice was thought to be predominantly mediated by melanopsin-based phototransduction, considering that the sleep response was abolished in mice lacking this photopigment, in particular during the dark phase. Given the spectral sensitivity of OPN4, this suggests that the blue portion of the light spectrum was mainly responsible for sleep induction. The authors offer an explanation for this, that given the green spectral sensitivity of the sleep-promoting response, mediation more likely arises from rods/cones rather than melanopsin, via M1 ipRGCs that are more dependent upon rod/cone input [25]. Thus, in the absence of melanopsin, the reduced sleep induction by light might result from coupling deficiencies and subsequent decreased functional ability of the rods and cones to transmit non-visual information through ipRGCs to the VLPO. In the future, the respective contribution of OPN4- versus rods/cones-based photoreception or either M1 versus non-M1 ipRGCs should be clarified using transgenic mouse models lacking one of each of the phototransduction pathways.

The study also provides additional information on the neural pathways involved in the photic regulation of sleep and alertness. Analysis of plasma corticosterone levels and gene induction in different brain areas in response to photic stimuli suggests that different neural routes mediate the opposing responses to blue/green wavelengths. Green light promotes sleep induction via non-M1 pRGCs, providing information to the sleep-promoting neurons of the VLPO, which in turn are known to inhibit histaminergic, noradrenergic, and serotoninergic arousal systems [26]. On the other hand, Pilorz et al. suggest that blue light enhances arousal through M1 pRGCs projecting to the SCN, resulting in elevated corticosterone via autonomic innervation of the adrenal glands, and possible subsequent activation of wake-promoting noradrenergic cells located in the locus coeruleus. This is comparable to the well-known pathway mediating melatonin suppression in response to light in the sense that it also involves signals relayed through the SCN and sympathetic nervous system. However, one would anticipate the involvement of additional arousal pathways, such as the melanopsin-innervated subparaventricular zone. This latter structure has direct projections to the dorsomedial hypothalamus, which in turn regulates the VLPO and the lateral hypothalamus, where the arousal hypocretinergic and sleep-promoting melanin-concentrating hormone (MCH) systems are located. Finally, in line with this, it might be of great interest to study diurnal rodent models, such as the African grass rat, *Arvicanthis*, in which the activation of the lateral hypothalamus, serotoninergic dorsal raphe and locus coeruleus correlate with the administration of light pulses [27].

## Blue Versus Green: A Model to Reconcile Research from Nocturnal and Diurnal Species?

**Fig 2:**
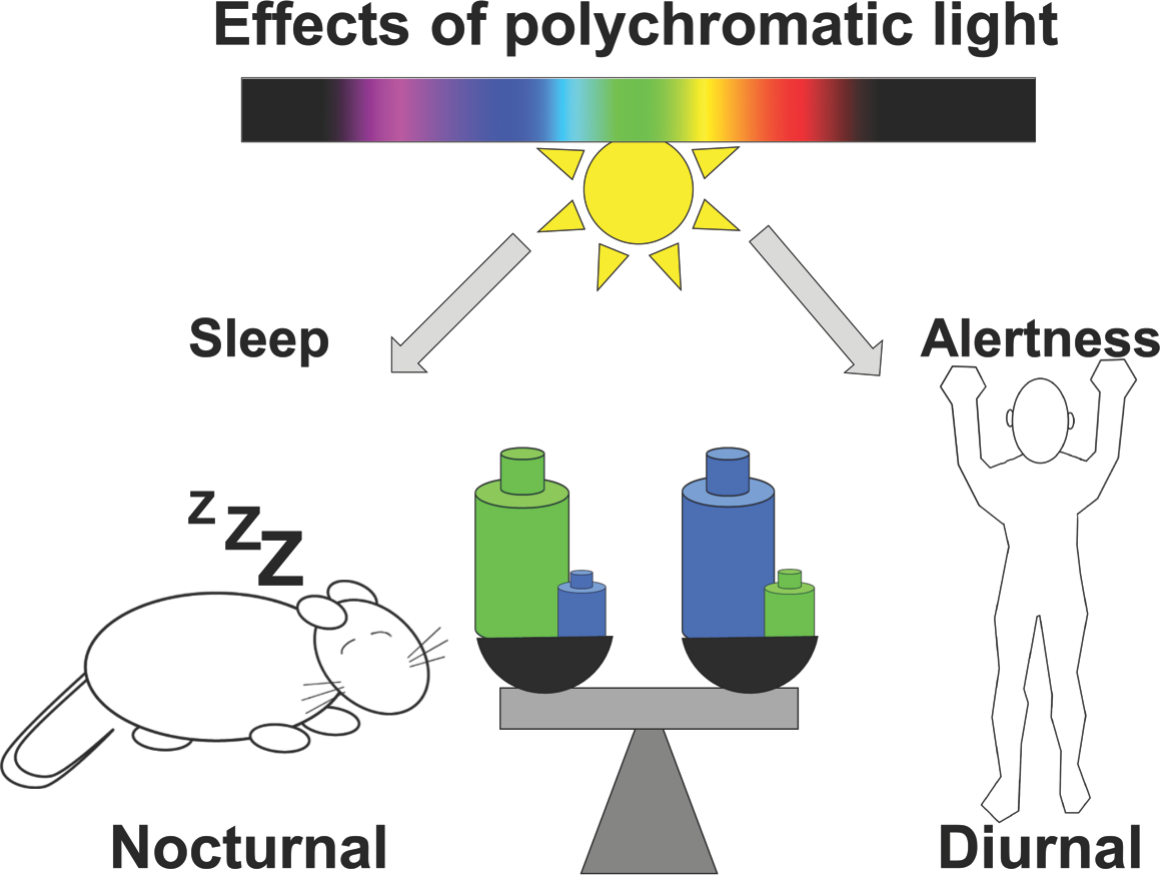
Light effects in diurnal and nocturnal species: A complex role of the spectral composition. Light is known to promote sleep in nocturnal species and alertness in day-active animals. Our hypothesis is that the opposite responses to natural polychromatic light may result from higher sensitivity of nocturnal species to green light, and diurnals to blue wavelengths, resulting in hypnogenic and alerting effects, respectively. This can be modeled as different blue and green weights on a balance, explaining either an alerting (higher sensitivity to blue) or sleep-promoting effect (greater sensitivity to green) of white light in diurnal and nocturnal species, respectively.

Light is known to exert opposite direct effects on sleep and waking in nocturnal and diurnal species. In fact, Pilorz and colleagues’ findings suggest that the photic regulation of sleep and alertness is far more complex, with their observations raising several questions. How does white polychromatic light promote sleep in nocturnal species when its effect is assumed to be primarily mediated by OPN4-based photoreception, i.e., by the blue portion of light spectrum? Another question is what non-visual role does green light play in humans? The observation that blue light exerts an alerting effect in nocturnal mice is consistent with human data on both alertness and melatonin suppression, and somehow reconciles nocturnality with diurnality within the mammalian kingdoms. Mice exposed to a light pulse normally display robust, though slightly delayed, sleep induction. According to Pilorz et al., the animals respond similarly under blue light, though with much greater latency. Given the duality between differing irradiance colors, the kinetics of white light’s effects on sleep induction may depend upon a competition between the sleep-promoting effects of longer wavelength (green) and the arousal stimulation of shorter (blue) wavelengths, with the latter eliciting a more immediate response. Applied to humans, one could explain the alerting effect of polychromatic light, as shorter wavelengths are far more efficient than longer wavelengths. Finally, one can hypothesize that the opposite responses to natural polychromatic light may reflect a higher sensitivity of nocturnal species to green, and diurnals to blue wavelengths, resulting in hypnogenic and alerting effects, respectively. This can be modeled as different blue and green weights on a balance, explaining either an alerting (higher sensitivity to blue) or sleep-promoting effect (greater sensitivity to green) of white light in diurnal and nocturnal species, respectively (Fig 2).

## Significance and Implications

We have known for some time that the influence of lighting environment on our physiology and behavior varies with irradiance levels, duration, and the time of day of light exposure. The spectral composition of light is another factor to consider, and almost all the attention has been focused on the blue color, as light enriched in blue wavelengths is known to be more efficient in mediating non-visual effects. However, the present study by Pilorz et al. reveals that the role of color is far more important and complex than previously thought and is a key parameter to take into account. Their findings in mice bring to the forefront a novel concept by which light can exert opposite effects on sleep and alertness, dependent upon wavelength composition. Indeed, their data suggest that, when exposed to white polychromatic light, the ratio of blue and green light within the overall spectral power will define the direction of the effect, promoting either sleep or alertness.

Translated to humans, these results reinforce the idea that blue light exerts a powerful alerting effect compared to green light [14,15,23,24]. This will open important avenues for both medical and broader societal applications. Light is currently used as a therapeutic tool, with an efficiency well-established for medical conditions such as circadian rhythm sleep disorders, seasonal affective disorders, and other depression subtypes [28]. Stimulating alertness or maintaining sleep using different wavelength compositions may lead to additional indications of light therapy as a complementary and innocuous treatment to help patients with insomnia symptoms or excessive daytime sleepiness. Considering societal implications, with the development of LED and progressive switching from fluorescent or other light devices to this newer technology, we are increasingly exposed to non-homogeneous spectra of light. Thus, the possibility to balance light wavelength composition in favor of alertness or sleep maintenance would allow the adaptation of our illuminated surroundings to our needs, with reservation to the fact that light can also have indirect effects through phase shifting of circadian rhythms. This type of spectral management could be applicable to many daily living conditions, far beyond simply the workplace or the home, allowing us to also better adapt to situations like transmeridian travel or shift-work, in addition to dealing with increasingly continuous screen exposure using modern media and other concomitants of life in modern society. Finally, Pilorz, et al.’s findings call for a greater understanding of melanopsin-based phototransduction and tells us that color wavelength is another aspect of environmental illumination that we should consider, in addition to photon density, duration of exposure, and time of day, as we move forward in designing the lighting of the future, aiming to improve human health and well-being.

## Abbreviations

ipRGCs: retinal ganglion cells
MCH: melanin-concentrating hormone
MWS: midwave-sensitive
NREM: non-rapid eye movement
OPN4: melanopsin
REM: rapid eye movement
SCN: suprachiasmatic nuclei
UVS: ultraviolet-sensitive
VLPO: ventrolateral preoptic area
